# Infliximab concentrations in two non-switching cohorts of patients with inflammatory bowel disease: originator vs. biosimilar

**DOI:** 10.1038/s41598-020-74235-1

**Published:** 2020-10-13

**Authors:** Ana Martínez-Feito, Luz Yadira Bravo-Gallego, Borja Hernández-Breijo, Jesús Diez, Laura García-Ramirez, Marta Jaquotot, Chamaida Plasencia-Rodríguez, Pilar Nozal, Araceli Mezcua, María Dolores Martín- Arranz, Dora Pascual-Salcedo

**Affiliations:** 1 Immuno-Rheumatology Group, La Paz University Hospital Institute for Health Research (IdiPAZ), Madrid, Spain; 2 Immunology Unit, La Paz University Hospital, Madrid, Spain; 3 Lymphocyte Pathophysiology in Immunodeficiencies Group, La Paz Institute for Health Research (IdiPAZ) and Center for Biomedical Network Research on Rare Diseases (CIBERER U767), Madrid, Spain; 4 Biostatistics Section, La Paz University Hospital, Madrid, Spain; 5 Unit of Inflammatory Bowel Disease, Gastroenterology Department. Innate Immunity Group, La Paz University Hospital Institute for Health Research (IdiPAZ), Madrid, Spain

**Keywords:** Immunological disorders, Immunotherapy, Gastroenterology, Inflammatory bowel disease

## Abstract

Biosimilars are replacing originator compounds due to their similar effectiveness, safety and pharmacokinetics. Our objective was to compare the differences in pharmacokinetics and clinical outcomes between the originator infliximab (Ifx) and the biosimilar CT-P13 in a patient cohort with inflammatory bowel disease (IBD). Our cohort study included 86 patients from a historical and a prospective cohort from the start of infliximab treatment to 22 weeks later. Serum infliximab, antidrug antibody levels and other serum biomarkers were measured at weeks 0, 2, 6, 14 and 22. Remission outcomes were evaluated at weeks 14 and 22. Drug levels were measured prospectively and analysed using MANOVA. Of the 86 patients, 44 (51%) and 42 (49%) were administered the originator and CT-P13, respectively. Originator trough levels were higher than the biosimilar trough levels (35 vs. 21, 20.1 vs. 11, 6.6 vs. 2.9 and 4.3 vs. 1.7 μg/mL at weeks 2, 6, 14 and 22, respectively). A post-hoc analysis demonstrated changes in mean serum drug levels over time (p < 0.001) and according to the drug employed (p = 0.001). At week 22, 13 (81%) patients administered the originator achieved clinical remission compared with 5 (19%) patients with the biosimilar (p = 0.02). None of the patients administered the originator withdrew from the treatment compared with 7 for the biosimilar. During the study, there were significant differences in serum infliximab levels between the originator and the CT-P13 in the patients with IBD. The clinical outcomes were influenced by the type of compound administered.

## Introduction

Inflammatory bowel disease (IBD) (consisting of Crohn’s disease [CD] and ulcerative colitis [UC]) is a chronic, relapsing–remitting inflammatory disorder of the gastrointestinal tract. IBD is influenced by several factors, including a dysregulated immune response^1^. Monoclonal antibody-mediated blockage of the proinflammatory cytokine tumour necrosis factor alpha (TNF α) is effective at reducing IBD in many cases, highlighting the key role TNF α plays in intestinal inflammation^2^. Since the introduction of anti-TNF drugs two decades ago, IBD treatment has significantly improved.

The patents for older anti-TNF drugs have either expired or are currently close to expiration. Biotech companies may copy and market these biological drugs, which, as with generics, could reduce the cost for patients and healthcare systems^3^. The European Medicines Agency defines the regulatory term “biosimilar” as “a biological medicinal product that contains a version of the active substance of an already authorized original biological medicinal product (reference medicinal product)”^4^.

As with other biological drugs, anti-TNF presents substantially high molecular heterogeneity inherent in its structure. In fact, the intrinsic complexity of these biological drugs and their biotechnology production in cell lines cannot be fully replicated. These compounds also present microheterogeneity, which entails differences in post-translational modifications such as glycosylation and phosphorylation^3^. We therefore cannot assume that the biological and pharmacological properties of the originator are identical to those of the biosimilar.

Despite the possibility of minor differences, the biosimilar must ultimately behave identically (in clinical terms) to the reference biological drug. In fact, major efforts have been undertaken to design clinical trials^5,6^ that demonstrate the clinical similarity of biosimilars. Regardless, there is a lack of in-depth studies that analyse the influence of variations in molecular structure on the “behaviour” of biosimilars.

In September 2013, the European Medicines Agency licensed CT-P13, the first biosimilar of infliximab, under the brand names Remsima and Inflectra. Since the introduction of these compounds in the European Union market in early 2015, most of the data from cohort studies of patients with IBD^7–11^ have supported the biosimilarity of CT-P13 and its reference product, with no significant differences in terms of effectiveness, safety or pharmacokinetics in either treatment-naive or switched patients^12^.

Differences in immunogenicity between the biosimilar and reference drug cannot be excluded a priori, and the antidrug antibody (ADA) production by the biosimilar (although largely predicted by that of the original drug) has been extensively studied^13–15^.

Therapeutic drug monitoring (TDM) is a clinical decision-making tool that enables dosage regimen adjustments based on laboratory measurements (typically drug concentrations in blood) to reach drug concentrations that are associated with the highest possible response rate^16^. TDM of biosimilars is performed identically using the same assays as those employed for the originator drugs^4,17^.

Since the introduction of biosimilars at our hospital in 2015, patients who have started a biological treatment with infliximab have received the biosimilar compound, in accordance with a management agreement with the Health Council of the Community of Madrid. The aim of this study was to compare circulating trough concentrations and immunogenicity development at treatment induction between the originator Remicade and the biosimilar CT-P13 in a patient cohort treated exclusively with either drug. We also analysed the clinical response of both cohorts.

## Materials and methods

### Patients and serum samples

The cohort study included 86 patients with IBD recruited from a cohort of patients treated with biological drugs at the IBD Unit of the Gastroenterology Department of La Paz University Hospital (Madrid). Since September 2015, all patients who have started infliximab treatment have been administered the biosimilar CT-P13, in accordance with an agreement between La Paz University Hospital and the Health Council of the Community of Madrid. We therefore compared two cohorts, the first of which was retrospective and included patients treated with the original compound Remicade who were recruited from 2010 to 2015. The second cohort was prospective and included patients who started infliximab treatment with the biosimilar CT-P13 from 2015 to 2018, when the data set was locked. All patients were diagnosed with Crohn’s disease (CD) or ulcerative colitis (UC), were included in the study from the start of their infliximab treatment and were monitored for at least 6 months. Nineteen patients had previously been administered another anti-TNF agent (adalimumab in all cases) but none were switched from the originator drug to the biosimilar during the study. All patients were administered intravenous infusions of 5 mg/kg of infliximab at weeks (W) 0, 2 and 6 and every 8 weeks thereafter. Of the total group, 44 patients were administered the originator Remicade, and 42 patients were administered the biosimilar CT-P13, either Remsima [CT-P13 (1)] or Inflectra [CT-P13 (2)].

Laboratory parameters were assessed during each infliximab infusion visit. Serum biomarker concentrations of C-reactive protein (CRP), albumin, platelets, haemoglobin, fibrinogen and erythrocyte sedimentation rate were recorded throughout the study period.

Serum infliximab and ADA samples taken at each infliximab infusion (weeks 0, 2, 6, 14 and 22) were stored at − 20 °C. Once all of the patients’ serum samples had been collected and after the database had been locked, the samples were unfrozen and measured.

The study was retrospectively approved by the Ethics Committee of La Paz University Hospital (PI: 2348) in April 2016. The overall study objective was to implement the TDM of TNF inhibitors in patients with IBD, which covered all patients who started infliximab therapy since 2010. The study will follow the national and international ethical protocols (Code of Ethics and Declaration of Helsinki) and will comply with the Spanish legal requirements for the maintenance of confidential data. All patients as well as healthy controls signed an informed consent document.

### Serum infliximab assay

Serum infliximab trough concentrations were measured by a capture ELISA commercial kit (MabTrack *Level Infliximab M2920*) developed by Sanquin (Amsterdam, The Netherlands) performed in an AP22 IF ELITE automated system supplied by Menarini Diagnostics.

This method captures infliximab by binding it to recombinant TNF immobilized onto the plate via a specific monoclonal antibody. Serum infliximab concentrations are then detected with a horseradish peroxidase-labelled anti-infliximab-idiotype antibody and tetramethylbenzidine as the chromogenic substrate. The drug concentration is then interpolated on a standard curve with known infliximab concentrations. This assay was used to measure the concentrations of the originator Remicade and the biosimilar CT-P13.

### Serum antidrug antibody assay

We assessed ADA levels using a drug sensitive in-house 2-site (bridging) ELISA, as previously described^18^. Cut-off values for the drug and ADA levels (in arbitrary units [AU]/mL) were established with a group of 250 healthy controls. The same assay was employed to investigate the development of ADA to the originator Remicade and to the biosimilar CT-P13.

### Clinical outcomes

The clinical response was evaluated from the medical records according to the gastroenterologist’s criteria (Dra. M. Jaquotot and Dra. MD. Martín-Arranz) supplemented with laboratory and, in some cases, endoscopic and image data as recommended by the 2010 and 2012 ECCO guidelines^19–22^. Differences in baseline clinical activity were assessed by CRP levels.

We considered the overall drug efficacy as complete, partial or non-responsive, which included (for patients with CD) both the luminal response and the perianal disease response. We defined clinical remission as achieving a complete response (based on the criteria of the 2010–2012 CD and UC guidelines) with the biological drug without requiring steroids. For CD^19,20^, a complete response was indicated by an assessment of clinical disease data by a gastroenterologist and objective data such as CRP and, where possible, imaging and endoscopy data. For UC, a complete response was indicated by a combination of clinical parameters (stool frequency ≤ 3/day and no bleeding)^21,22^. No clinical remission was considered when there was a partial response, loss of response (needing infliximab dosage intensification or the addition of concomitant treatment) or no primary response.

We also analysed treatment survival at W22 between the two groups, as well as the CRP based-remission (defined as a CRP level ≤ 5 mg/L), among the patients with CRP levels > 5 mg/L at baseline^23,24^. Both remission outcomes were analysed at W14 and W22.

### Statistical analysis

The descriptive statistics are presented as mean and standard deviation (SD) or median and interquartile range (IQR), depending on normality. The qualitative variables are listed as absolutes, and the relative frequencies are expressed as percentages. We assessed the differences in clinical and biological characteristics between the two groups using Pearson’s chi-squared test or Fisher’s exact test for the categorical variables and with the Mann–Whitney U test or Kruskal–Wallis for the continuous quantitative variables. Given that we grouped patients with CD and UC, we evaluated the clinical response as a dichotomous variable (clinical remission/no clinical remission). We analysed the serum drug levels prospectively using a repeated measures analysis of variance or a multivariate analysis of variance (MANOVA), along with the Greenhouse–Geisser test. For the statistically significant results, we employed the Bonferroni Post Hoc test to examine all possible differences between the time points. We performed a general linear model and an interpatient effect test to adjust for diagnosis, sex, smoking status, previous biological therapy and immunomodulator (IM) use as confounding factors. We then analysed drug survival using Kaplan–Meier curves and compared the groups using the log-rank test. For all analyses, we employed SPSS 20.0 software and GraphPad Prism 6 (San Diego, CA, USA) and considered p-values < 0.05 statistically significant.

## Results

### Patient characteristics

The study included 86 patients with IBD, 59 (69%) of whom had CD and 27 (31%) of whom had UC. The originator Remicade was administered to 44 (51%) patients, and the biosimilar CT-P13 was administered to 42 (49%) patients: 17 (40%) patients were administered Remsima and 25 (60%) were administered Inflectra. The patients’ demographic characteristics are shown in Table 1. There were no significant differences in baseline clinical activity between the two groups. Sixty-seven patients (78%) were treatment *naïve* to anti-TNF and other biological therapies, and 45 patients (52%) were women. Concomitant IM use (79%) was similar in the two groups with azathioprine the most widely used IM (94% patients), and 10 patients (12%) were treated with corticosteroids.

**Table 1:** Demographic and baseline characteristics of 86 patients with inflammatory bowel disease.

Characteristics	Total population (n = 86)	Remicade group (n = 44)	Biosimilar group (n = 42)	p
Age at diagnosis, years^a^	32.5 (23–44.75)	32 (25–43)	33 (18.5–46.5)	0.8
Age, years^a^	43 (33–55)	41.5 (33–50.5)	48.5 (32.2–57.7)	0.3
Female^b^	45 (52%)	23 (52%)	22 (52%)	1
**Diagnosis**^b^				0.36
CD	59 (69%)	28 (64%)	31 (74%)	
UC	27 (31%)	16 (36%)	11 (26%)	
**Smoking status**^b^				0.47
Never smoker	25 (35%)	14 (34%)	11 (36%)	
Current smoker	21 (24%)	10 (24%)	11 (36%)	
Previous smoker	26 (30%)	17 (42%)	9 (29%)	
Previous biological therapy^b^	19 (22%)	10 (23%)	9 (21%)	1
Baseline patient weight, kg^b^	68 (58–77)	64 (55–80)^c^	68 (58–77)	0.7
**Montreal classification of CD**^b^				
L1 (isolated ileal disease)	17 (28%)	7 (25%)	10 (32%)	0.5
L2 (isolated colonic disease)	6 (10%)	5 (18%)	1 (3%)	0.09
L3 (ileocolonic disease)	29 (49%)	13 (46%)	16 (52%)	0.8
L4 (concomitant UGI disease)	7 (12%)	3 (11%)	4 (13%)	1
B1	32 (37%)	12 (43%)	11 (36%)	0.6
B2	16 (19%)	8 (29%)	8 (26%)	1
B3	10 (12%)	6 (21%)	4 (13%)	0.5
A1	6 (7%)	2 (7%)	4 (10%)	0.4
A2	33 (38%)	18 (64%)	15 (49%)	0.7
A3	20 (23%)	8 (29%)	12 (39%)	0.6
**Montreal classification of UC**^b^				1
E1	0	0	0	
E2	8 (9%)	5 (31%)	3 (27%)	1
E3	18 (21%)	11 (69%)	7 (64%)	1
Perianal disease^b^	20 (34%)	9 (32%)	11 (35%)	0.6
Serum albumin, g/L^a^	4 (3.6–4.2)	4.1 (3.6–4.3)	4 (3.6–4)	0.5
CRP at baseline, mg/L^a^	5 (1.4–22.2)	3 (1.5–21.3)	6.7 (1.4–23)	0.7
CRP > 5 at baseline, mg/L^b^	43 (50%)	19 (44%)	24 (56%)	0.3
Immunomodulator^b^	57/72 (79%)	34/41 (83%)	23/31 (74%)	0.4

*CD* Crohn’s disease, *CRP* C-reactive protein, *UC* ulcerative colitis, *UGI* upper gastrointestinal.

^a^Median (interquartile range).

^b^n (%).

^c^Data of 35 patients.

### Serum infliximab trough levels at induction phase

**Figure 1:**
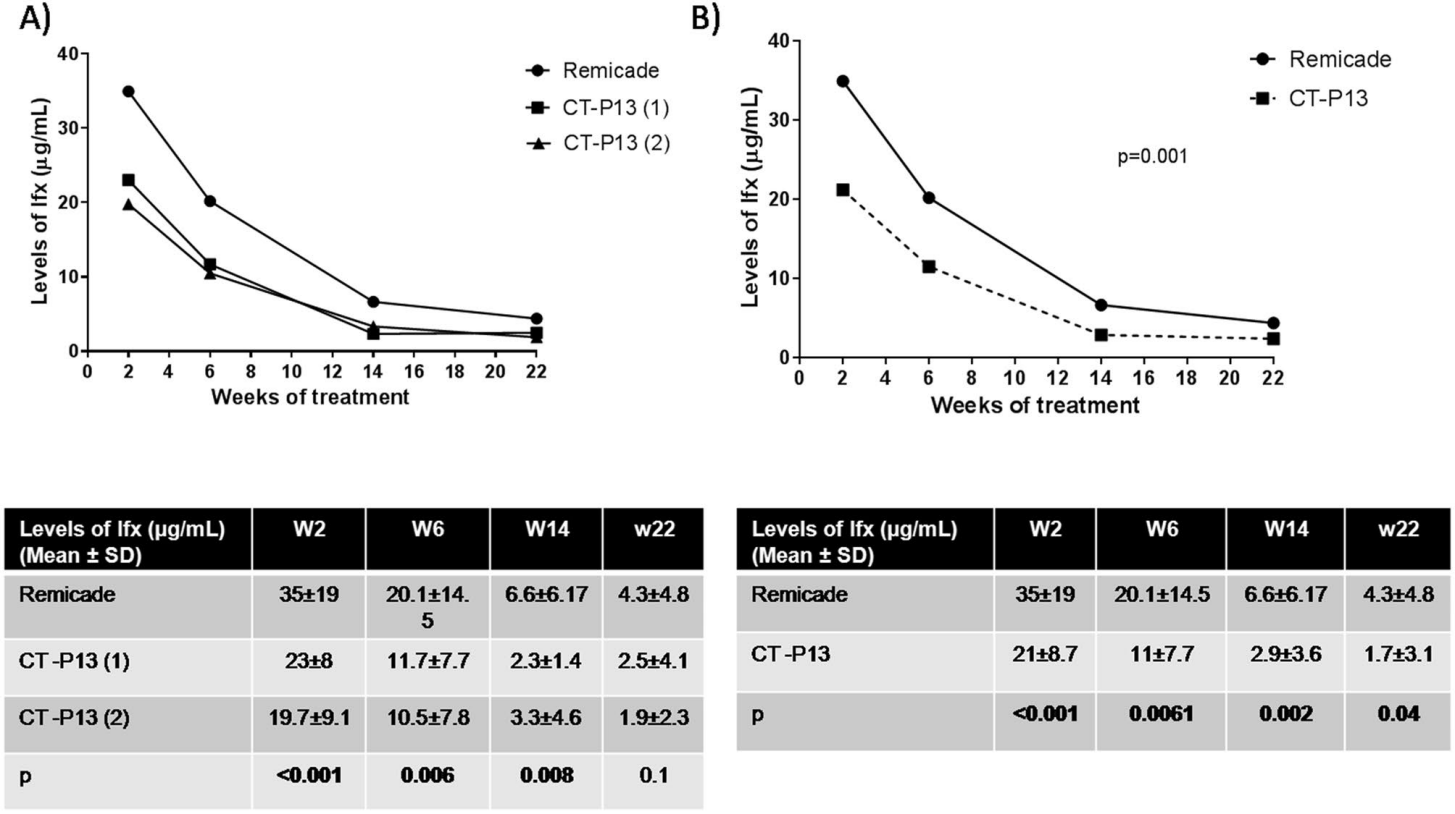
Mean serum trough infliximab levels (μg/mL) during the induction phase (weeks 2, 6, 14 and 22) in the patients with IBD. (**A**) patients who started treatment with the originator (Remicade) or a commercial presentation of the biosimilar CT-P13(1) or CT-P13(2); (**B**) patients who started treatment with the originator (Remicade) or the biosimilar (CT-P13). The table shows the mean and standard deviation (SD) of the serum infliximab levels at each studied time point. Kruskal–Wallis test, p < 0.05. Greenhouse–Geisser test, p < 0.05.

We assayed 430 serum samples (5 samples per patient) and compared the median serum infliximab levels of the originator Remicade and the two commercial CT-P13 presentations (Remsima [CT-P13 (1)] and Inflectra [CT-P13 (2)]). There were statistically significant differences at all time points except W22: p < 0.001 at W2; p = 0.006 at W6; p = 0.008 at W14; and p = 0.1 at W22 (Fig. 1A).

The post-hoc analyses showed a significant difference in serum infliximab levels between Remicade and Inflectra and between Remicade and Remsima at all studied time points, but there were no differences between Remsima and Inflectra, which was expected because they are the same compound (p = 1 at all time points). We therefore grouped the data from patients administered one or the other commercial presentation and re-compared the serum trough levels for the originator and biosimilar. The MANOVA test for prospective measurements showed that the serum trough levels for the originator were higher than those of the biosimilar at all time points (35 vs. 21 μg/mL at W2; 20.1 vs. 11 μg/mL at W6; 6.6 vs. 2.9 μg/mL at W14; and 4.3 vs. 1.7 μg/mL at W22). There were statistically significant differences at all time points (p < 0.001 at W2; p = 0.001 at W6; p = 0.002 at W14; and p = 0.04 at W22) (Fig. 1B). The Greenhouse–Geisser test showed changes in mean serum drug levels over time (p < 0.001) and according to the drug employed (p = 0.001) but not with diagnosis (p = 0.35), sex (p = 0.78), smoking status (p = 0.75) and previous biological therapy (p = 0.36). Pairwise comparisons showed significant differences in serum infliximab (originator vs. biosimilar) concentrations at each time point, adjusted by the Bonferroni test (p < 0.01).

We defined a serum drug concentration of 3–8 μg/mL at W22 as an optimal concentration associated with a good clinical response^25^. Fewer patients treated with CT-P13 had serum trough levels within this optimal range (22% with CT-P13 vs. 30% with Remicade) (p = 0.02).

### The immunomodulator’s effect on circulating drug levels

A high percentage (79%) of patients were administered an IM concomitantly with the infliximab therapy (95% azathioprine and 5% methotrexate). Given that IM has a well-known effect on serum drug concentrations^26,27^, we performed the MANOVA test and included the use or non-use of concomitant IM to evaluate the possible interaction of this variable with serum infliximab concentrations. At all studied time points, serum infliximab levels were higher in the patients with an IM than in the patients without IM, regardless of infliximab treatment. However, the multivariate analysis showed that the only variable with a significant effect on serum infliximab levels was the type of infliximab (p = 0.004), while concomitant IM use had no significant effect (p = 0.175).

### Immunogenicity

Development of immunogenicity during the induction phase was similar for both groups (8 patients developed ADA with the originator, and 7 patients developed ADA with the biosimilar). However, one patient treated with CT-P13 had detectable ADA in serum by W6 (data not shown).

### Serological biomarkers

We investigated whether the percentage of patients achieving CRP values ≤ 5 mg/L at W14 and W22 was related to the administered drug (originator or biosimilar). Of the 86 patients, 43 had CRP levels > 5 mg/L at baseline. More patients treated with the originator Remicade achieved CRP-based remission than with the biosimilar CT-P13, although the differences were not statistically significant (at W14, 56% vs. 44% of patients for the originator and biosimilar, respectively [p = 0.1]; at W22, 53% vs. 46%, respectively [p = 0.6]).

**Figure 2:**
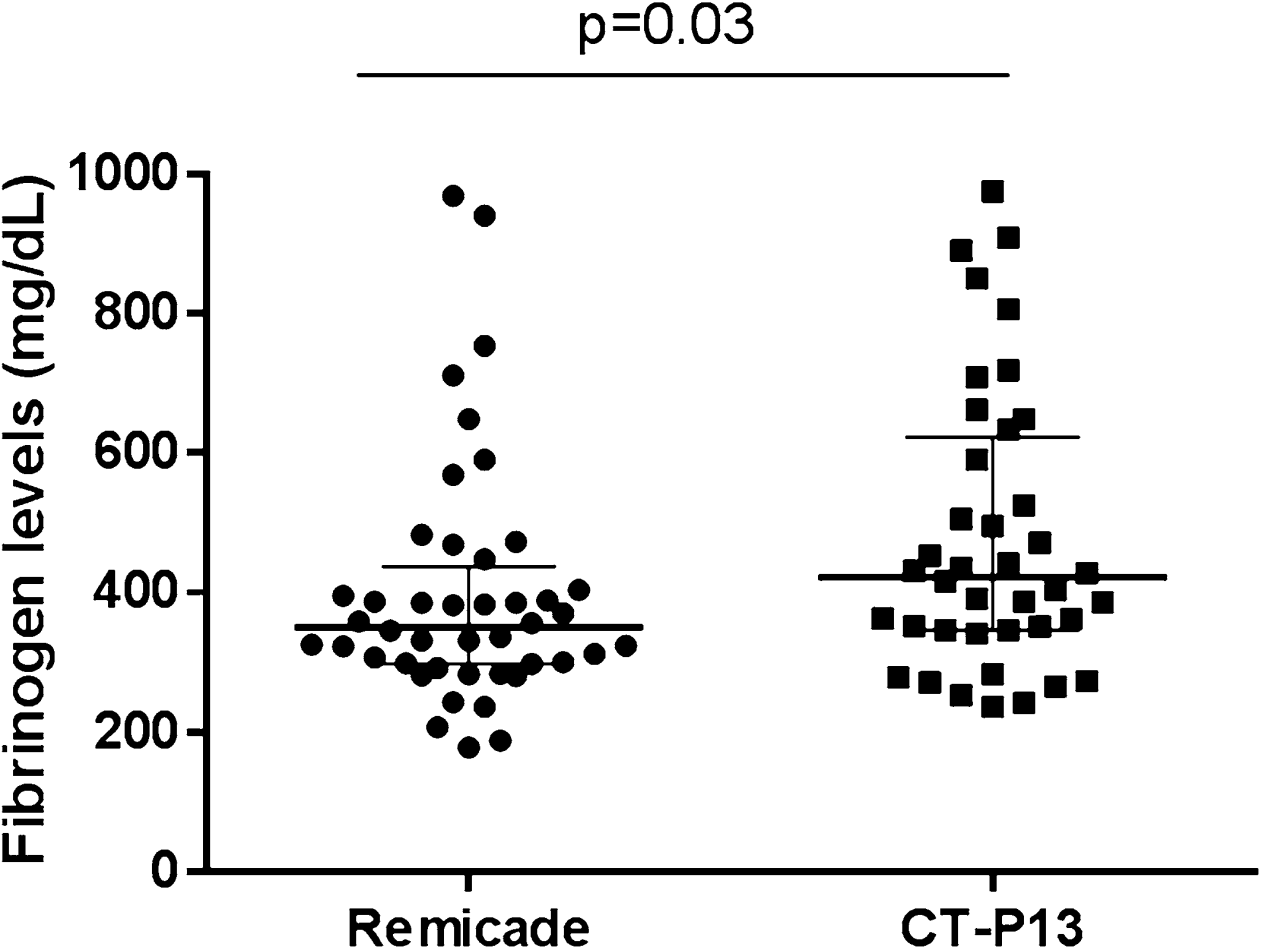
Serum fibrinogen levels (mg/dL) at week 22 in the patients with IBD undergoing therapy with Remicade or biosimilar (CT-P13). Mann–Whitney test, p < 0.05.

When we analysed other blood serum parameters, we found statistical differences only in fibrinogen levels at W22 between the patients treated with the originator Remicade and those treated with biosimilar CT-P13 (median 350.5 [IQR 289.2–437] mg/dL vs. 478.1 [IQR 346–623] mg/dL; p = 0.03) (Fig. 2).

### Clinical remission

Clinical remission data were available for 80 patients at W14 and for 81 patients at W22. At W14, only 16 patients had achieved clinical remission, 11 (68.8%) of whom were undergoing originator Remicade therapy and 5 (31.2%) of whom were undergoing biosimilar CT-P13 therapy (p = 0.3).

At W22, 13 (81.2%) of the 16 patients who had achieved clinical remission were treated with the originator, while 3 (18.8%) were treated with the biosimilar (p = 0.02). At this time point, the estimated risk of achieving clinical remission was 4.9-fold higher for the patients treated with the originator Remicade (95% CI 1.28–18.87) than for the patients treated with the biosimilar CT-P13.

We further investigated whether concomitant IM use affected clinical remission. For the patients treated concomitantly with IM (n = 63), 32.4% of the patients treated with the originator Remicade achieved clinical remission versus 10.3% of the patients who underwent biosimilar CT-P13 therapy (p = 0.06). For the patients not treated concomitantly with IM (n = 15), 25% of the patients treated with the originator Remicade achieved clinical remission vs. 0% of the patients who underwent biosimilar CT-P13 therapy (p = 0.46). In both situations, more patients undergoing originator Remicade therapy achieved clinical remission, regardless of IM use.

Accordingly, patients who did not achieve clinical remission had higher fibrinogen levels than the patients who achieved clinical remission at W14 and W22 (372 [310–490] vs. 337 [248.5–387] mg/dL [p = 0.06] at W14; and 385 [312–590] vs. 346 [252–346] mg/dL [p = 0.05] at W22).

### Treatment survival

**Figure 3:**
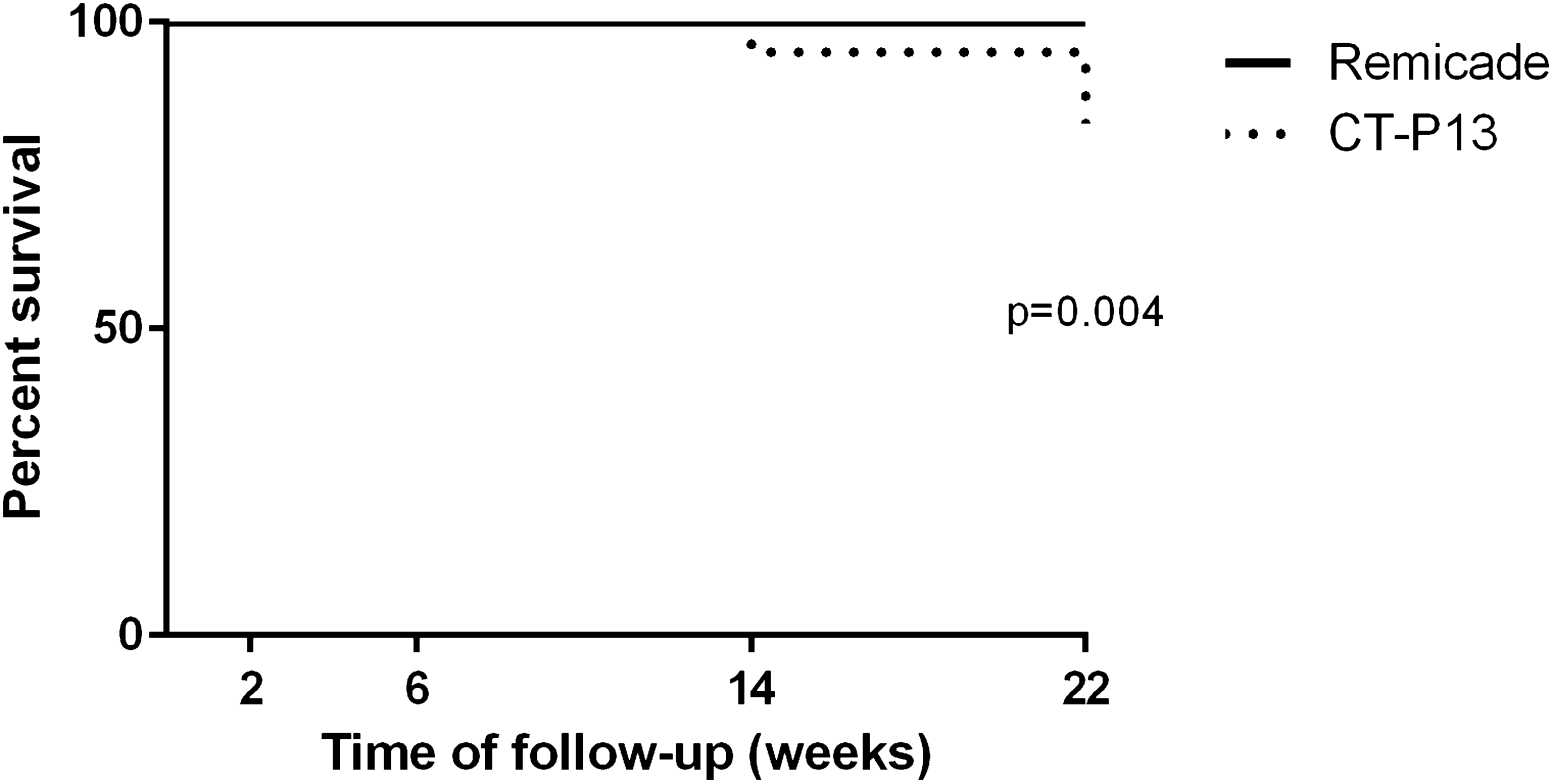
Drug survival during the 22 weeks according to the therapy with Remicade or biosimilar (CT-P13). Log-rank (Mantel Cox) test, chi squared test; p < 0.05.

By the end of the study (W22), none of the patients treated with the originator molecule had withdrawn from the therapy, whereas 7 (17%) patients (3 with CD and 4 with UC) treated with the biosimilar withdrew at W22 (3 due to secondary failure, 3 due to primary failure and 1 for surgery) (Fig. 3). The percentage of patients who withdrew from the treatment was not associated with having undergone therapy with another anti-TNF agent (adalimumab) before the study (10 [23%] patients underwent Remicade therapy, and 9 (21%) patients underwent CT-P13 therapy).

## Discussion

To the best of our knowledge, the present study is the first to compare serum trough levels of the originator infliximab and its biosimilar CT-P13 and to correlate these levels with clinical outcomes in a population of non-switched patients with IBD.

Most postlaunch studies of biosimilars have focused on demonstrating equivalence in terms of clinical efficacy, effectiveness and safety based on a comprehensive comparability exercise^28^. The interchangeability of biosimilars allows them to be employed instead of the original compound, while producing the same expected clinical effect^28^. However, there are few in-depth analyses of the implications of the small differences in molecular structure on the biosimilar’s pharmacokinetic/pharmacodynamic properties^29^.

A recently published study showed that differences in the glycosylation profile (e.g., fucosylation) of monoclonal antibodies results in differences in FcγR-IIIa binding affinities and antibody-directed cellular cytotoxicity (ADCC) activity. Remicade, which has higher levels of afucosylated glycans (100.9%) than Inflectra (77%), binds with higher affinity to FcγR-IIIa and exhibits stronger ADCC activity, leading to better efficacy in IBD. Furthermore, the difference in ADCC activity follows this trend, with 99.8% and 50.3% relative activity for Remicade and Inflectra, respectively^30^.

This study presents data from two comparable cohorts of non-switched patients with IBD treated with the originator infliximab (Remicade) or with the infliximab biosimilar CT-P13. Our results suggest that, at standard doses, CT-P13 might not be completely interchangeable with the originator Remicade. Serum levels of the biosimilar CT-P13 were lower than those of the originator Remicade during the 22 weeks of treatment induction, and more patients treated with the biosimilar CT-P13 discontinued treatment. All patients had received the same dose at the same interval, and there were no significant demographic differences between the two treatment groups.

Most published articles on this topic have focused only on the clinical outcomes of CT-P13^7,8,31,32^ treatment, while other articles have compared patient groups who switched from the originator to the biosimilar^9–11,17,33,34^, obtaining a positive correlation between the two drugs in terms of efficacy and safety.

However, few studies have compared serum trough levels of Remicade vs. CT-P13. A study published by Schulze et al.^35^ of 119 patients with IBD (33 treated with CT-P13 and 86 treated with Remicade) reported identical serum levels and no significant differences in drug immunogenicity between Remicade and CT-P13 during the first 38 weeks of therapy. However, the authors did observe a clear tendency towards lower CT-P13 levels (perhaps due to the low number of patients at weeks 22, 30 and 38). Furthermore, the authors did not evaluate any association with clinical outcomes.

A study by Ratnakumaran et al.^10^ examined the efficacy and tolerability of CT-P13 treatment in 69 patients with IBD compared with 53 anti-TNF treatment-*naïve* patients who started with the infliximab originator and were monitored for 12 months. In contrast to our results, the authors found similar remission rates (defined as CRP levels < 5 mg/L) and treatment responses with the two drugs. We cannot rule out the possibility that the different baseline characteristics of the patients in the authors’ two groups played a role in the comparison of clinical outcomes between the groups. As with our study, the authors found that lower circulating drug levels were associated with higher rates of loss of response.

Schmitz et al.^17^ performed TDM on a small group of patients with rheumatoid arthritis to monitor the switch from the originator infliximab to the biosimilar infliximab. The authors found no differences in drug levels after switching from the originator to the biosimilar; however, they did not indicate the time between the last Remicade infusion and the first CT-P13 infusion. Given that ELISA assays can detect both Remicade and CT-P13^36^, it is possible that lower serum CT-P13 levels were nor found after switching because the authors were measuring both drugs simultaneously present in serum. This artifact might have also affected the comparison of drug levels in other studies on switching^33^, given that 5 mg/kg of administered Remicade can remain in circulation for longer than 16 weeks^18^, thereby potentially affecting the detection of CT-P13. Moreover, none of these studies compared the serum levels of switched patients with those of patients who continued with the originator therapy. Furthermore, our data cannot be compared with the SECURE study (and similar studies)^33^, because that study included only patients in remission (who are expected to maintain fairly stable drug concentrations), while our study included any patient who met the clinical criteria for starting CT-P13 as the first treatment.

There were no differences in immunogenicity in our cohort, given that an equal number of patients treated with Remicade or with CT-P13 developed antidrug antibodies over the 22 weeks of the study, which is consistent with previously published studies^5,12–15^. However, one of the patients treated with the biosimilar CT-P13 developed ADA earlier (at W6) than any other patient undergoing originator Remicade therapy.

In clinical practice, concomitant IM use is very frequent. IMs have a beneficial effect on the clinical response and have been associated with a reduced risk of developing ADA^26,27^ and with higher circulating levels of infliximab^37^. All analyses performed in this study were adjusted for the concomitant use of IM as a confounding factor, which showed no significant influence on serum infliximab levels.

The correlation between the presence of serum infliximab and the clinical response in IBD^38–41^ has been widely demonstrated. Our study showed that patients treated with CT-P13 had lower serum trough infliximab levels and that a lower proportion of these patients achieved clinical remission at W22 compared with patients treated with the originator Remicade (18% vs. 81%, respectively). Our results also show significant differences in treatment survival between the drugs; none of the patients undergoing Remicade therapy withdrew from the study, whereas 7 (17%) patients undergoing CT-P13 treatment withdrew. A recently published study by Yazici et al. reported higher discontinuation rates for patients with rheumatoid arthritis and IBD treated with CT-P13^42^, highlighting the fact that patients treated with CT-P13 withdraw earlier that those treated with infliximab.

Although more patients undergoing Remicade therapy achieved CRP-based remission (as determined by CRP levels), the differences compared with CT-P13 were not statistically significant. However, we found differences in the acute phase reactant fibrinogen, which has been described as one of the best serological markers for detecting endoscopic activity in CD^43^ and has been correlated with active disease^44–46^. In our cohort, serum fibrinogen levels were higher in patients undergoing CT-P13 therapy than in the patients undergoing Remicade therapy. Furthermore, fibrinogen values were associated with not achieving clinical remission, regardless of the type of treatment.

The main limitations of this study are the methods to evaluate the clinical assessment and the retrospective design of one of the two cohorts. In this sense, faecal calprotectin measurements and clinical evaluation with imaging or endoscopy were not routinely performed in both cohorts. Moreover, although the clinical remission was evaluated by two specialized gastroenterologist, the guideline recommendations used were outdated and quite vague to assess the clinical activity. Other limitations include the small size of the cohort and the lack of differentiation between CD. Nevertheless, all patients were administered the same drug dose with the same administration interval, which is required for comparing serum trough levels between two groups. A significant strength of our study is that it compares both treatments in similar groups without switching between originator and biosimilar, thereby avoiding the nocebo effect and other possible bias when evaluating the clinical outcomes.

In summary, our findings show significant differences in serum trough infliximab levels between the originator Remicade and the biosimilar CT-P13 during the treatment induction phase in the patients with IBD. The type of administered compound also affects clinical outcomes such as disease remission and drug survival. The pharmacokinetics of the two compounds appear to differ; the biosimilar is cleared sooner than the originator, possibly due to reduced binding to the Fcγ receptor, as was described by Kang et al.^30^. Further studies might be needed to investigate the influence of small structural changes (post-translational modifications) that could lead to the differences observed between the two drugs. Accordingly, if the two drugs have different clearance rates, the advantage of using biosimilars in terms of cost-effectiveness might need to be reviewed. Presumably, higher doses of the biosimilar are required to achieve the same effects as the originators. Caution is advised, however, when generalising these findings to other biological agents. Further long-term comparative studies including broader cohorts are needed.
